# Genome-wide identification and analysis of high-affinity nitrate transporter 2 (*NRT2*) family genes in rapeseed (*Brassica napus* L.) and their responses to various stresses

**DOI:** 10.1186/s12870-020-02648-1

**Published:** 2020-10-09

**Authors:** Jiafeng Tong, Thomas C. Walk, Peipei Han, Liyu Chen, Xinjie Shen, Yinshui Li, Chiming Gu, Lihua Xie, Xiaojia Hu, Xing Liao, Lu Qin

**Affiliations:** 1 Oil Crops Research Institute of Chinese Academy of Agricultural Sciences/Key Laboratory of Biology and Genetics Improvement of Oil Crops of the Ministry of Agriculture and Rural Affairs, Wuhan, 430062 P. R. China; 2 Tropotech LLC, St. Louis, MO 63141 USA; 3 Institute of Agriculture Science in Jiangsu Coastal Area, Yancheng, 224002 P. R. China; 4 Innovative Center of Molecular Genetics and Evolution, School of Life Sciences, Guangzhou University, Guangzhou, 510006 P. R. China

**Keywords:** High-affinity nitrate transporter, *NRT2*, Rapeseed, Various stresses

## Abstract

**Background:**

High-affinity nitrate transporter 2 (*NRT2*) genes have been implicated in nitrate absorption and remobilization under nitrogen (N) starvation stress in many plant species, yet little is known about this gene family respond to various stresses often occurs in the production of rapeseed (*Brassica napus* L.).

**Results:**

This report details identification of 17 *NRT2* gene family members in rapeseed, as well as, assessment of their expression profiles using RNA-seq analysis and qRT-PCR assays. In this study, all *BnNRT2.*1 members, *BnNRT2.2a* and *BnNRT2.4a* were specifically expressed in root tissues, while *BnNRT2.7a* and *BnNRT2.7b* were mainly expressed in aerial parts, including as the predominantly expressed *NRT2* genes detected in seeds. This pattern of shoot *NRT* expression, along with homology to an Arabidopsis *NRT* expressed in seeds, strongly suggests that both *BnNRT2.*7 genes play roles in seed nitrate accumulation. Another rapeseed *NRT*, *BnNRT2.5 s*, exhibited intermediate expression, with transcripts detected in both shoot and root tissues. Functionality of *BnNRT2s* genes was further outlined by testing for adaptive responses in expression to exposure to a series of environmental stresses, including N, phosphorus (P) or potassium (K) deficiency, waterlogging and drought. In these tests, most *NRT2* gene members were up-regulated by N starvation and restricted by the other stresses tested herein. In contrast to this overall trend, transcription of *BnNRT2.1a* was up-regulated under waterlogging and K deficiency stress, and *BnNRT2.5 s* was up-regulated in roots subjected to waterlogging. Furthermore, the mRNA levels of *BnNRT2.7 s* were enhanced under both waterlogging stress and P or K deficiency conditions. These results suggest that these three *BnNRT2* genes might participate in crosstalk among different stress response pathways.

**Conclusions:**

The results presented here outline a diverse set of *NRT2* genes present in the rapeseed genome that collectively carry out specific functions throughout rapeseed development, while also responding not just to N deficiency, but also to several other stresses. Targeting of individual *BnNRT2* members that coordinate rapeseed nitrate uptake and transport in response to cues from multiple stress response pathways could significantly expand the genetic resources available for improving rapeseed resistance to environmental stresses.

## Background

Nitrogen (N) is an essential plant macronutrient that is often limited in availability, which leads to millions of metric tons of nitrogenous fertilizer being applied at significant cost to agricultural fields worldwide [1, 2]. Furthermore, excessive application of N fertilizer degrades the environment through pollution of water and air, where it also contributes to global warming. Therefore, reducing the requirements for N fertilizer through improvements in crop N use efficiency (NUE) is a worthwhile research objectives for crop breeders and plant scientists [3].

For most plant species, uptake, assimilation, and translocation processes are vital early steps for successful acquisition and utilization of N from soil sources. Nitrogen assimilation requires the reduction of nitrate (NO_3_^−^) to ammonium, followed by incorporation of ammonium into amino acids, which depends on the acquisition and transport of inorganic N sources and transfer of amino acids and proteins between multiple plant organs [4]. Since NO_3_^−^ is the most abundant inorganic N in soils [1], NO_3_^−^ uptake and translocation, therefore, play crucial roles in NUE.

The composition of N in the soil is affected by many factors, including mositure, temperature, wind, soil type and pH, which has led to large variations in nitrate concentrations among soil environments [3, 5]. In order to cope with the heterogeneity of NO_3_^−^ in the soil, plants have evolved two NO_3_^−^ absorption systems, namely a low-affinity transport system (LATS), and a high-affinity transport system (HATS), which are mediated by nitrate transporter 1 (NRT1) and nitrate transporter 2 (NRT2), respectively [6]. Overall, NRT2 activity supplants NRT1 activity as N availability diminishes. In one set of comparable results, expression in *Xenopus oocytes* has led to *Km* value estimates of 5.9 mM for the *Arabidopsis* LATS transporter *AtNRT1.2* and 30 μM for the barley HATS transporter *HvNRT2.1* [7, 8]. This nearly 200 fold variation in nitrate affinity suggests that HATS *NRT2* may be key targets in efforts to improve NUE, especially under low N availability conditions.

Seven high-affinity nitrate transporters (*NRT2.1*-*NRT2.7*) have been identified in *Arabidopsis thaliana* (L.) [6, 9]. To date, *AtNRT2.1*, *AtNRT2.2* and *AtNRT2.4* have been implicated in root NO_3_^−^ uptake [10, 11]. Interestingly, AtNRT2.1 plays a predominant role in NO_3_^−^ absorption, while AtNRT2.2 is capable of compensating for lost NO_3_ uptake when AtNRT2.1 is incapacitated [12]. For its part, AtNRT2.4 not only acts in NO_3_^−^ absorption, but also contributes to loading NO_3_^−^ in shoot phloem vessels [11]. Another high-affinity nitrate transporter in *Arabidopsis*, AtNRT2*.5*, is up-regulated under N limiting conditions in both shoots and roots of mature plants, where it supports efficient uptake of nitrate, as well as, nitrate loading into the phloem during nitrate remobilization [13]. *AtNRT2.7* has been reported to localize on the vacuolar membrane, it plays specifics roles in seed nitrate accumulation [14]. Neither *NRT2.3* nor *NRT2.6* have been implicated in NO_3_^−^ uptake or translocation. Similar activities by homologues of *Arabidopsis NRT2* genes in crop species have been partially confirmed, though efforts have been limited. Work with rice has revealed five *NRT2* genes, including *OsNRT2.3a*, which was found to play a key role in root to shoot nitrate translocation under N limiting conditions [15].

Rapeseed (*Brassica napus* L.) is a globally important oil crop that is cultivated widely for production of vegetable oil, biofuels and industrial products, such as lubricants and surfactants [16, 17]. As an allotetraploid, the *Brassica napus* (genome A_n_A_n_C_n_C_n_) is a product of genome doubling in a hybridization between *Brassica oleracea* (L.) (genome C_o_C_o_) and *Brassica rapa* (L.) (genome A_r_A_r_) ~ 7500 years ago [18]. This suggests that the *Brassica napus* genome might harbor multiple homologs for individual *NRT2* members in *Arabidopsis* [18]. Rapeseed requires substantial amounts of N, and, as a result, is extremely sensitive to changes in soil N availability [19]. Reducing the input of N fertilizer while maintaining yields of rapeseed will, therefore, likely entail improving the NUE of rapeseed. Furthermore, rapeseed is sensitive to various environmental stresses, such as macronutrient deficiencies, waterlogging and drought stresses, these will lead to the nutrients imbanlance, influence the N metabolism or other metabolic pathways, finally suppress the growth and yield of rapeseed [20–25]. It’s necessary to understand the adaptive patterns of *NRT2* family genes in response to these stresses which commonly appeared in the production of rapeseed. Thus, the aim of this study is (i) to identify all *NRT2s* family members in the *Brassica napus* genome, (ii) analyze the genomic and transcriptomic characteristics of *BnNRT2s* family members, including exon-intron organization, conserved motifs, phylogeny, gene duplication, tissue specific expression, and their responsive profiles under various stresses, and (iii) determine core gene members contributing to NO_3_^−^ absorption and transportation under N limiting conditions or other environmental stresses. Overall, this report reveals the characteristics of *NRT2* genes in rapeseed and also provides candidate gene resources for improving NUE and reducing N fertilizer applications to rapeseed fields.

## Results

### Genome-wide identification and bioinformatic analysis of rapeseed *NRT2* family genes

To identify *NRT2* genes members in rapeseed, the amino acid sequences of AtNRT2s were used to perform a BLAST search of the Brassica Database (BRAD), which returned 17 genes identified as *NRT2* family members in rapeseed, as listed in Table 1. These *BnNRT2* genes were labelled according to their top *Arabidopsis* matches as *BnNRT2.1 s* (7 genes), *BnNRT2.2 s* (2 genes), *BnNRT2.3 s* (2 genes), *BnNRT2.4 s* (2 genes), *BnNRT2.5 s* (2 genes) and *BnNRT2.7 s* (2 genes). No homologues of *AtNRT2.6* were identified in the rapeseed genome. According to the BRAD physical map, the identified *BnNRT2* gene members mapped onto 9 chromosomes (chrA02, chrA06, chrA08, chrA09, chrA10; chrC02, chrC03, chrC08, chrC09), with *BnNRT2* genes distributed almost equally in the A and C genomes (Fig. S1). For example, *BnNRT2.5a* and *BnNRT2.5b* mapped to chrC03 and chrA08, respectively, while *BnNRT2.7a* and *BnNRT2.7b* were respectively mapped to chrC02 and chrA02 (Fig. S1). This provide undeniable evidence for the presence of more *NRT2s* gene members in rapeseed than in *Arabidopsis*, which is due to genome multiplication in the hybridization between *B.oleracea* (genome C_o_C_o_) and *B. rapa* (genome A_r_A_r_). Gene duplication analysis in rapeseed indeed found that of 14 identified BnNRT2 duplicated pairs, 10 gene pairs were derived from whole-genome duplication, and 4 genes pairs were derived from transposed duplication (Table S1).

**Table 1:** Molecular characterization of the NRT2 family proteins in rapeseed

No.	Gene name	Gene ID	CDS (bp)	Amino acids (aa)	MY	PI	Subcellular localization
1	*BnNRT2.1a*	BnaC08g02430D	1395	464	50,555.41	8.79	PM
2	*BnNRT2.1b*	BnaAnng40490D	1062	353	38,542.64	9.16	PM
3	*BnNRT2.1c*	BnaAnng35750D	1062	353	38,491.53	9.14	PM
4	*BnNRT2.1d*	BnaA06g04560D	1383	460	50,267.16	8.98	PM
5	*BnNRT2.1e*	BnaA06g04570D	1395	464	50,665.68	8.94	PM
6	*BnNRT2.1f*	BnaA09g49050D	1266	421	45,571.37	9.14	PM
7	*BnNRT2.1 g*	BnaC08g43380D	1392	463	50,319.18	8.86	PM
8	*BnNRT2.2a*	BnaC08g43370D	1509	502	54,398.46	8.91	PM
9	*BnNRT2.2b*	BnaA09g49040D	1521	506	54,827.93	9.02	PM
10	*BnNRT2.3a*	BnaC09g54030D	1443	480	52,018.08	8.49	PM
11	*BnNRT2.3b*	BnaA10g13570D	1443	480	52,013.20	9.01	PM
12	*BnNRT2.4a*	BnaAnng28170D	1182	393	43,761.98	8.96	C (SP)
13	*BnNRT2.4b*	BnaC09g35990D	1371	456	50,062.26	9.33	M
14	*BnNRT2.5a*	BnaC03g56990D	1497	498	54,128.70	9.11	PM
15	*BnNRT2.5b*	BnaA08g24500D	1500	499	54,174.72	9.01	PM
16	*BnNRT2.7a*	BnaC02g05530D	1509	502	53,454.55	7.61	V
17	*BnNRT2.7b*	BnaA02g02200D	1455	484	52,062.10	7.54	V

*PM* plasma membrane, *C* cytoplasmic, *SP* signal peptide, *M* Mitochondrial, *V* Vacuolar

The length of BnNRT2 proteins range from 353 (BnNRT2.1b and BnNRT2.1c) to 506 (BnNRT2.2b) amino acid residues (Table 1). The percent identity between BnNRT2s (Table S2) ranged from 26.9% (BnNRT2.1b and BnNRT2.7a) to 98.8% (BnNRT2.5a and BnNRT2.5b). The minimum PI value calculated was 7.54 for BnNRT2.7b, which means that all BnNRT2s are basic proteins. Subcellular localization predictions indicate that most BnNRT2s localize on plasma membranes, except for BnNRT2.4a, which was predicted as a cytoplasmic protein with a signal peptide, BnNRT2.4b, which was predicted as a mitochondrial protein, and both BnNRT2.7 s, which were predicted as vacuolar proteins (Table 1). This variety in predicted subcellular localization suggests that BnNRT2s, like AtNRT2s, perform different transporter functions. The results of transmembrane helix predictions also support this conclusion, with individual BnNRT2s harboring 8 to 10 transmembrane helices (Fig. S2).

**Fig. 1:**
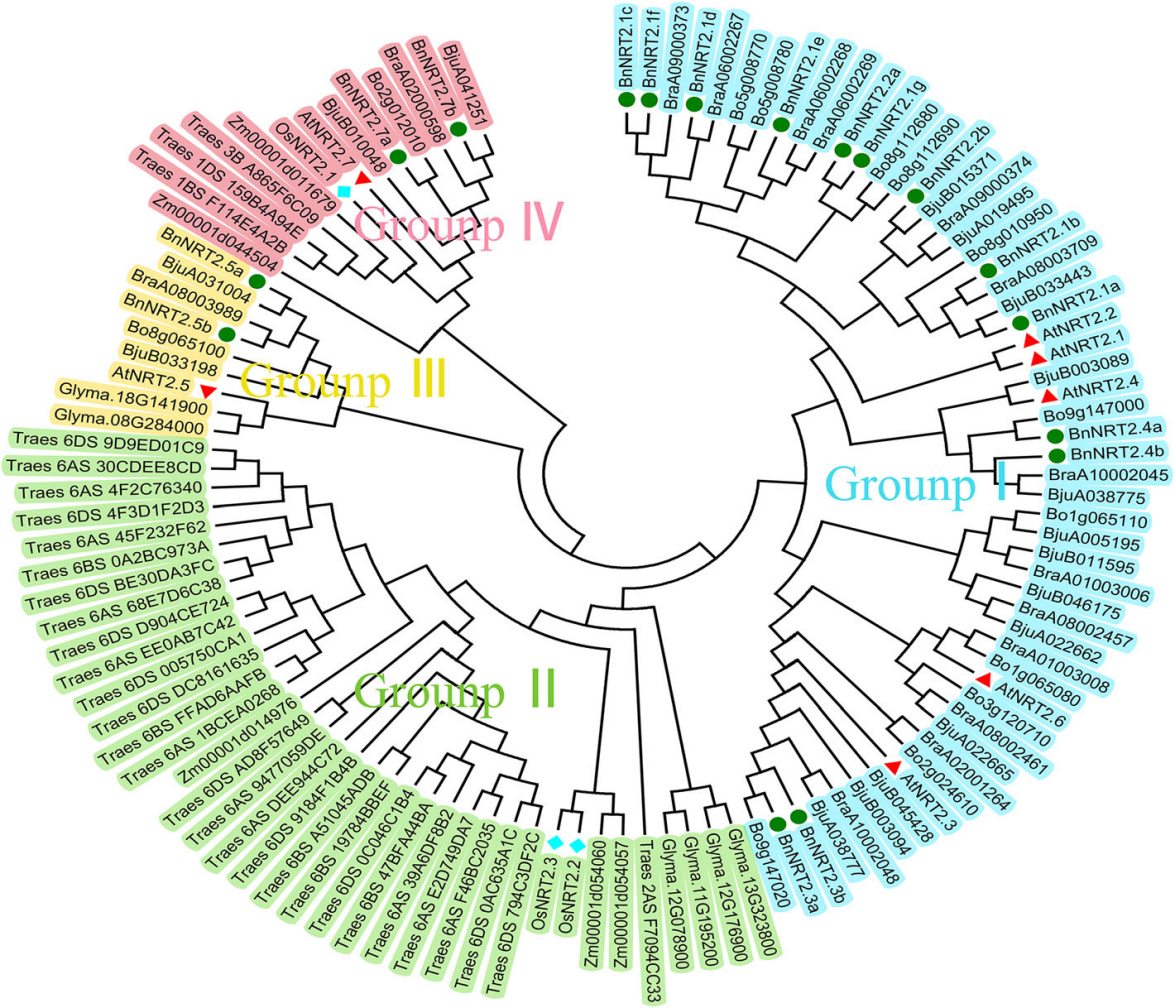
Phylogenetic relationships of NRT2 family proteins in diverse species. A total of 114 protein sequences were subjected to multiple sequence alignment in ClustalW prior to constructing a phylogenetic tree using the Neighbor-Joining method in MEGA 6.06. Different groups are labeled by different colors. The aligned NRT2 proteins originating from *A.thaliana*, *B.napus* and *O.sativa* are marked by triangles, circles and squares, respectively

To understand the phylogenetic relationships between NRT2 proteins in rapeseed and other species, an unrooted phylogenetic tree of NRT2 family members was constructed. As shown in Fig. 1, the tree was divided into four groups named groups I-IV. Among these four groups, BnNRT2.1 s, BnNRT2.2 s, BnNRT2.3 s and BnNRT2.4 s were distributed in group I, while BnNRT2.5 s and BnNRT2.7 s were distributed in groups III and IV, respectively, which further indicates that functional divergence has occurred between BnNRT2.5 s and BnNRT2.7 s. Comparisons of the analyzed species revealed that rapeseed NRT2 proteins align closely with their homologs from *A. thaliana,* but not with NRT2s from *O. sativa,* a monocotyledon with NRT2 protein members that fell mainly into group II.

**Fig. 2:**
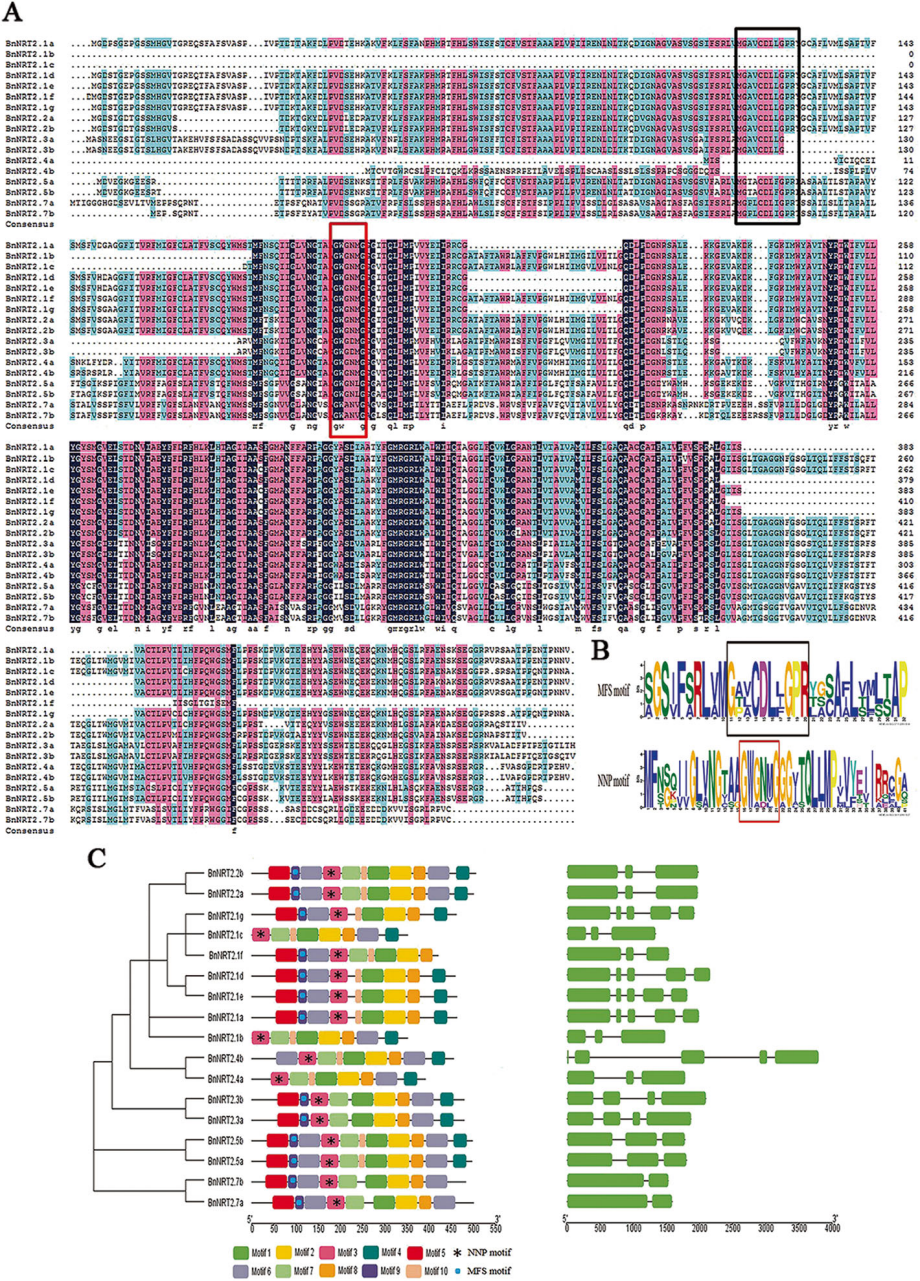
Gene structures and conserved domains in the NRT2 family protein of rapeseed. **a** A multiple sequence alignment of BnNRT2 family proteins constructed using DNAMAN software. The black frame and red frame represent the domain of MFS signature sequences and NNP signature sequences, respectively. **b** Amino acid sequences of the motif conserved across rapeseed NRT2s proteins, which contain signature sequences of MFS and NNP domains. The bigger the font size, the more likely the amino acid is at that location across all BnNRT2s proteins; **c** Boxes with different colors represent different conserved motifs, with the MFS and NNP motifs being denoted by circles and asterisks in the boxes, and a gene structure map of *BnNRT2*s was correspondingly constructed in GSDS2.0. Green boxes indicate exons, and black lines represent introns

The multiple sequence alignment results of BnNRT2 amino acid sequences, as performed in DNAMAN, showed that NRT2 proteins are likely nitrate/nitrite porter family (NNP) members of the Major Facilitator Superfamily (MFS), with both MFS (G-x-x-x-D-x-x-G-x-R) and NNP (G-W/L-G-N-M/A-G) signatures identified in BnNRT2 sequences (Fig. 2a). Weblogo outputs revealed that nearly all of the BnNRT2 protein members have a highly conserved NNP motif (G-W-G/A-N/D-M/L/V-G). The MFS motif (G-A/T/P-V/L/A-C-D-L-L/I/F-G-P-R), in contrast, was not found in BnNRT2.1b, BnNRT2.1c, BnNRT2.4a and BnNRT2.4b (Fig. 2a, b). Further analysis of BnNRT2 amino acid sequences in the MEME web app with the maximum number of motifs set to 10 revealed that all of the identified BnNRT2 members contain the conserved domain labelled as “motif 1-motif 2-motif 8” (Fig. 2c).

Gene structure analysis of *BnNRT2s* showed that *BnNRT2.2 s*, *BnNRT2.3 s*, *BnNRT2.5 s* and *BnNRT2.7 s* have the same number of introns and exons (Fig. 2c). Within contrast, *BnNRT2.4a and BnNRT2.4b* lack the first two exons (Fig. 2c), which might be indicative of functional divergence. What’s more, the gene structures of *BnNRT2.1* members can be divided into two types: 1) those with four introns and five exons (*BnNRT2.1a*, *BnNRT2.1d*, *BnNRT2.1e* and *BnNRT2.1 g*); and 2) those with two introns and three exons (*BnNRT2.1b*, *BnNRT2.1c* and *BnNRT2.1f*) (Fig. 2c).

### Analysis of putative cis-acting regulatory elements (CREs) in the promotor regions of *BnNRT2* family genes

**Fig. 3:**
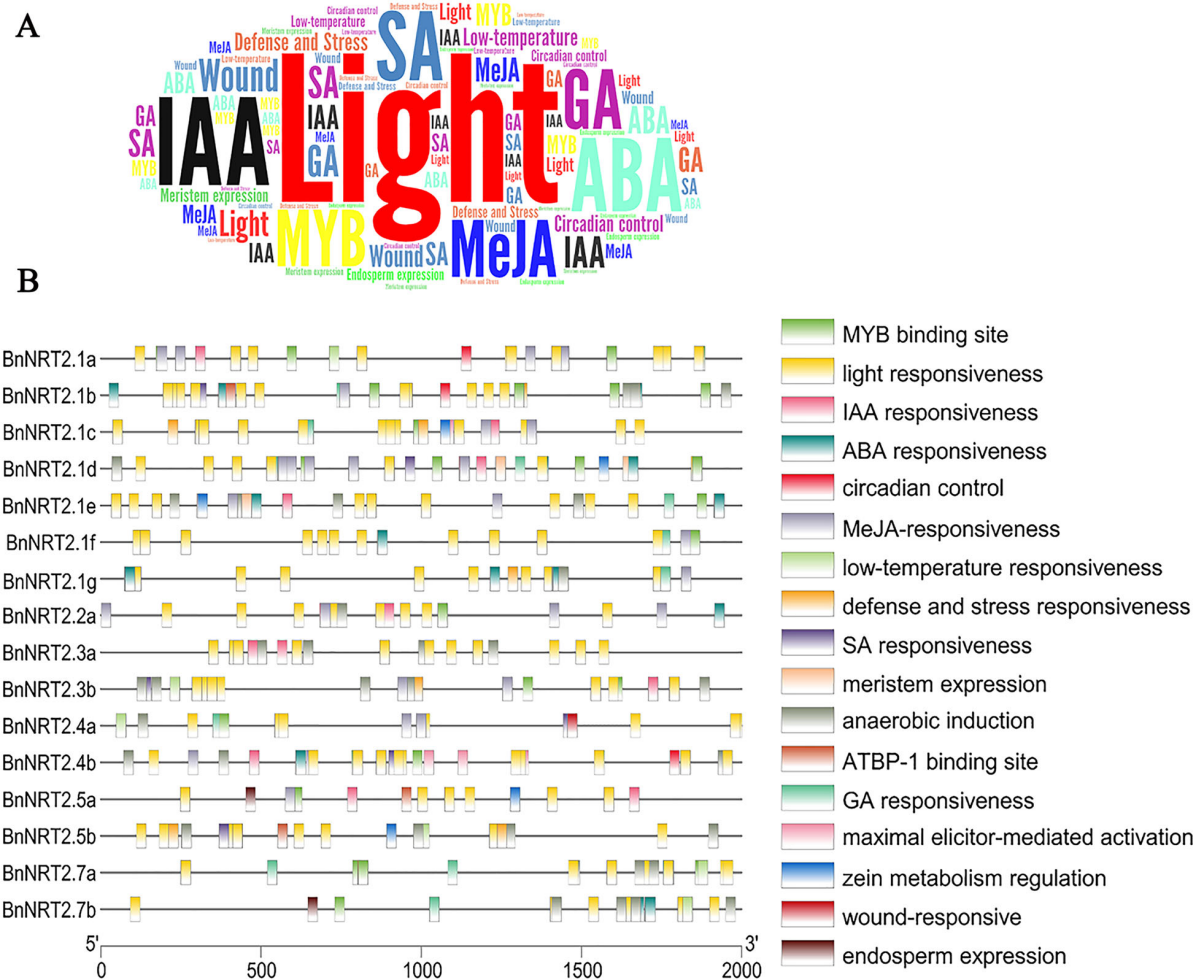
Identification of putative cis-acting regulatory elements (CREs) in the promoter regions of *BnNRT2* family genes. **a** The abundance of CREs in the promotor regions (2000 bp genomic sequences upstream the start codon) of *BnNRT2s* were determined in the WordArt program. Font size increases with the abundance of CREs. **b** Genomic distribution of CREs in promotor regions of *BnNRT2s*. Boxes filled with different colors represent different CREs

Transcriptional expression is regulated in large part through binding of transcription factors (TFs) to cis-acting regulatory elements (CREs). To gain insight into potential regulation of *BnNRT2*s, we performed CREs analysis on the 2000 bp region upstream of the start codon for each *BnNRT2*. As indicated in Fig. 3a, the most abundant CREs in all of the analyzed *BnNRT2* promotor regions were light responsive elements, followed by several hormone-related elements and MYB binding sites. To be specific, the CREs of rapeseed *NRT2* genes can be divided into four categories, namely MYB binding sites, abiotic stress responsive elements, hormone-related elements, growth and development regulative elements (Fig. 3b).

MYB proteins are multifunctional TFs that are involved in many plant processes [26]. MYB binding sites were found in all *BnNRT2* promoter regions, except those upstream of *BnNRT2.1 g*, *BnNRT2.3a* and *BnNRT2.5b* (Fig. 3b). Light responsive elements were found in abundance among the promoter regions of all the *BnNRT2* gene members, while low-temperature responsive elements were also found in the promoter regions of *BnNRT2.1a, BnNRT2.3b, BnNRT2.4a, BnNRT2.5b* and *BnNRT2.7 s* (Fig. 3b). The broader category of abiotic stress responsive elements, which refers to light responsiveness, low-temperature responsiveness, defense and stress responsiveness, anaerobic induction and wound-responsive, was represented extensively throughout *BnNRT2* promoter regions (Fig. 3b). In addition, hormone-related elements were well represented among *BnNRT2* promoter regions, with ABA, MeJA, SA, GA, and IAA responsive elements widely identified in the promotor regions of rapeseed *NRT2* genes (Fig. 3b). In contrast, growth and development regulatory elements were more sparsely distributed among Bn*NRT2* promoter regions. The identified growth and development CREs included circadian control related elements occurring in the promotor regions of *BnNRT2.1a, BnNRT2.1b* and *BnNRT2.4b*, meristem expression related elements occurring in the promotor regions of *BnNRT2.1b, BnNRT2.1d* and *BnNRT2.1e*, and endosperm expression related elements occurring only in the promotor regions of *BnNRT2.5a* and *BnNRT2.7b* (Fig. 3b).

### Tissue specific expression of rapeseed *NRT2* family genes

To explore the tissue specific expression profiles of *BnNRT2* genes, samples for RNA-seq and qRT-PCR analyses were collected from various tissues throughout the rapeseed lifecycle. Specifically, RNA-seq was performed with pistil, stamen, ovule, stem, leaf, root, silique, and pericarp samples, while qRT-PCR tests were run on flower, stem, junior leaf, senior leaf, root, seed and pericarp samples from adult plants. Early expression was also tested for *BnNRT2.5a*, and *BnNRT2.5a* in qRT-PCR tests performed with root and leaf samples at seedling stage of rapeseed.

**Fig. 4:**
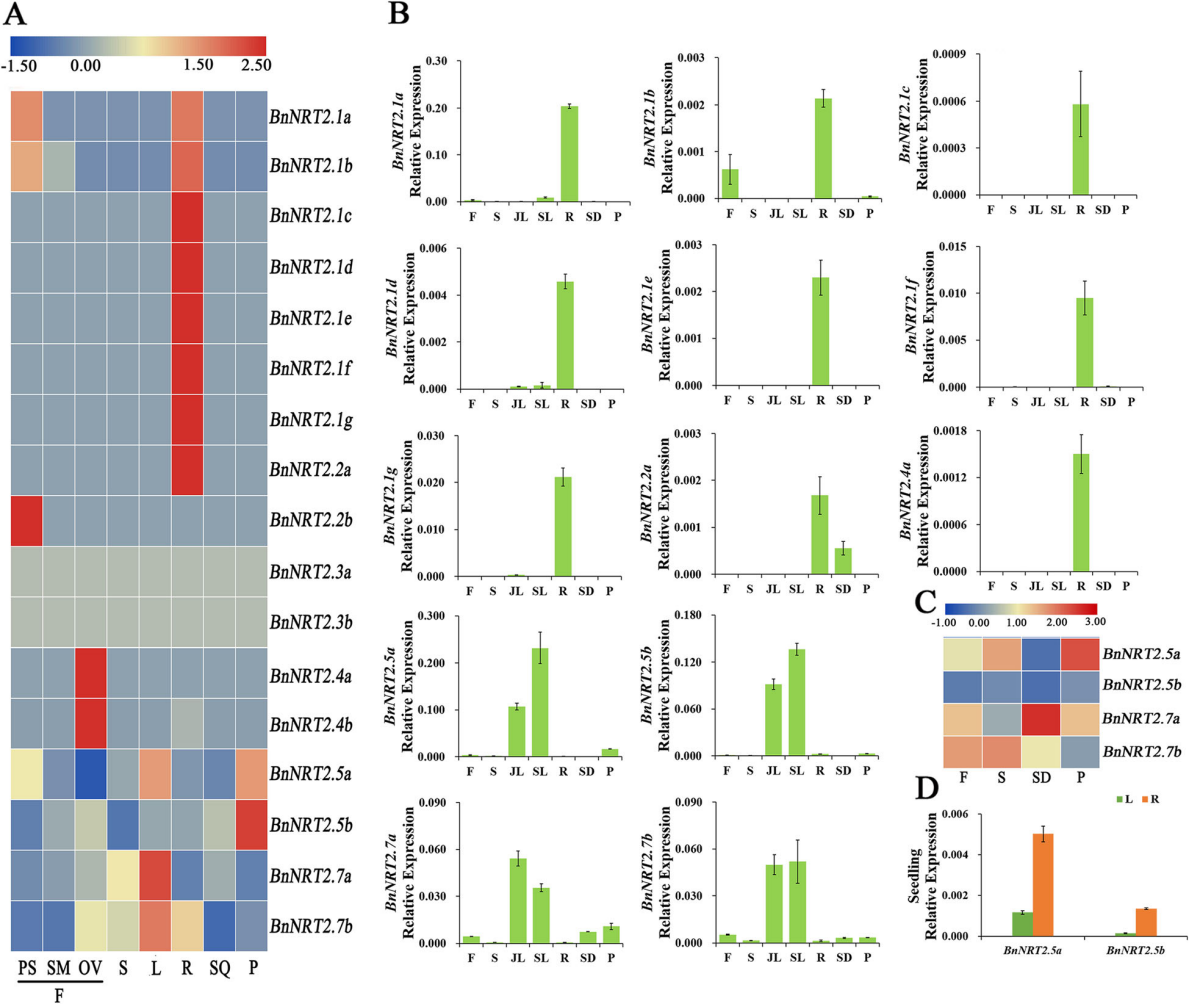
Tissue specific expression profiles of *BnNRT2* family genes as determined in RNA-seq and qRT-PCR assays. **a** Heat maps of relative expression from RNA-seq analysis of *BnNRT2* expression in eight different tissues. PS: pistil, SM: stamen, OV: ovule, S: stem, L: leaf, R: root, SQ: silique, P: pericarp. **b**-**d** Summary of tissue specific expression of *BnNRT2* genes determined in qRT-PCR assays. **b** Expression of *BnNRT2*s of different tissues in adult plants at different growth stages. Each tissue of adult plants were collected from field plots supplemented with N supplied as 180 kg/hm^2^ urea, in detail, junior leaf (JL), senior leaf (SL) and root (R) were collected at vegetative growth stage (namely over-wintering stage), flower (F) and stem (S) were collected at flowering stage, then seed (SD) and pericarp (P) were collected at reproductive growth stage. **c** The expression of *BnNRT2.5 s* in seedling tissues (21-days-old rapeseed seedlings). Rapeseed seedlings were reared in hydroponics cultures supplied with normal N treatment (9.5 mM N), L: leaf, R: root. **d** Expressions of *BnNRT2.5 s* and *BnNRT2.7 s* in the flower (F), stem (S), seed (SD) and pericarp (P) though qRT-PCR assays were further delineated in heat maps

Combined results from RNA-seq and qRT-PCR analyses revealed that in adult plants, *BnNRT2.1a-g*, *BnNRT2.2a* and *BnNRT2.4a* were mainly expressed in the root, with *BnNRT2.1a* being the most abundant root *BnNRT2*, and *BnNRT2.1b* transcripts abundant in both roots and flowers, especially pistils (Fig. 4a). Further observation of *BnNRT2* expression in qRT-PCR assays (Fig. 4b, c) largely confirmed the results from RNA-seq analysis (Fig. 4a). Notably, all *BnNRT2.1* genes were predominantly expressed in roots in both assays. Meanwhile, among shoot expressed *BnNRT2*s, *BnNRT2.5* and *BnNRT2.7* family members were primarily expressed in leaf tissue (Fig. 4b), though they were also obviously detectable in flowers, stems and pericarps, as well as, in seeds for *BnNRT2.*7 family members (Fig. 4c). Further qRT-PCR assays of *BnNTR2.5* tissue specific expression profiles conducted with hydroponically reared seedlings also returned detectable expression for both *BnNRT2.5a* and *BnNRT2.5b*, though shoot expression was much lower for these genes in seedlings than in adult plants to the point that expression was higher in roots than in shoots (Fig. 4d).

### *BnNRT2* family genes respond to N deprivation in the field

**Fig. 5:**
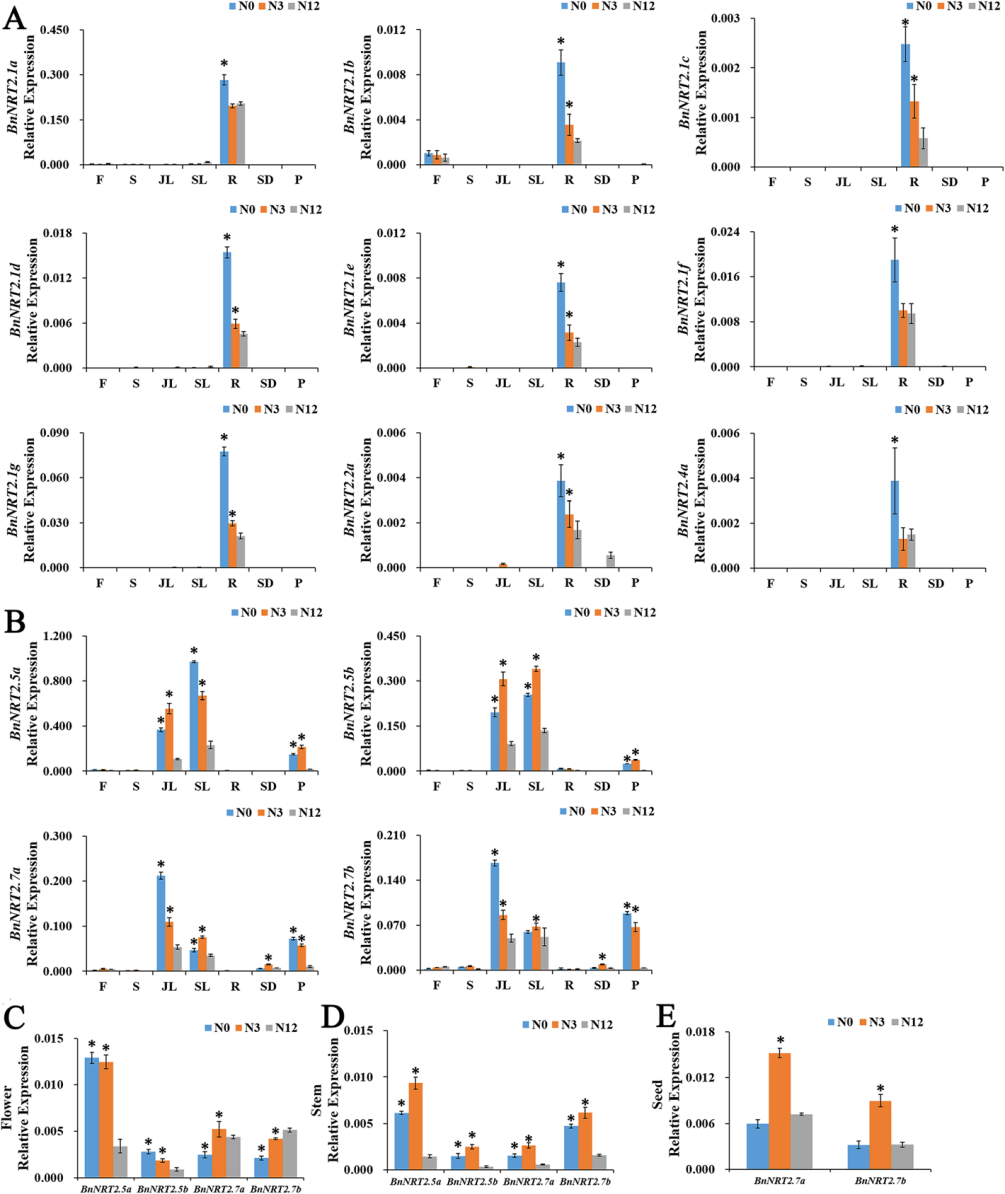
Responses of *BnNRT2* expression profiles to alterations in N supply as determined in qRT-PCR assays. **a** Responses of root specific *BnNRT2* genes (*BnNRT2.1* s, *BnNRT2.2a* and *BnNRT2.4a*). **b** Responses of shoot specific *BnNRT2* genes (*BnNRT2.5* s and *BnNRT2.7* s). **c** Responses of *BnNRT2.5* s and *BnNRT2.7* s in flower. **d** Responses of *BnNRT2.5* s and *BnNRT2.7* s in stem. **e** Responses of *BnNRT2.7 s* in seed. For these assays, rapeseed was cultivated in the field plots supplied with 180, 45 or 0 kg/hm^2^ N. The rapeseed plants grown in 180 kg/hm^2^ N-supplied plots were regarded as the control plots at each sampling time. Asterisks mark significant differences in expression between control plots and plots supplied 45 or 0 kg/hm^2^ of urea (*P* < 0.05)

To investigate the possible roles of *NRT2*s in rapeseed responses to N deprivation, the expression of *BnNRT2* family genes was assayed in various rapeseed tissues from plants grown in field plots producing significant N effects on biomass among urea application treatments (Fig. S3A, B). The results show dramatic up-regulation of *BnNRT2.1 s*, *BnNRT2.2a* and *BnNRT2.4a* in roots (Fig. 5a), and the expressions of *BnNRT2.5 s* and *BnNRT2.7 s* were significantly up-regulated in leaves, stems and pericarps of rapeseed plants responding to N deprivation (Fig. 5b). In addition, the abundance of *BnNRT2.*5 transcripts also significantly increased in flowers of rapeseed plants responding to N deprivation (Fig. 5c). The responses of *BnNRT2.7* s to N deprivation ran counter to the responses observed for other *BnNRT2* members, with expression peaking not in plants grown in the lowest N supply treatment plots, but rather in plants reared in intermediate or high N supply plots (Fig. 5c, d, e).

### Expression profiles of *BnNRT2* family genes in response to macronutrient deficiency in hydroponics

**Fig. 6:**
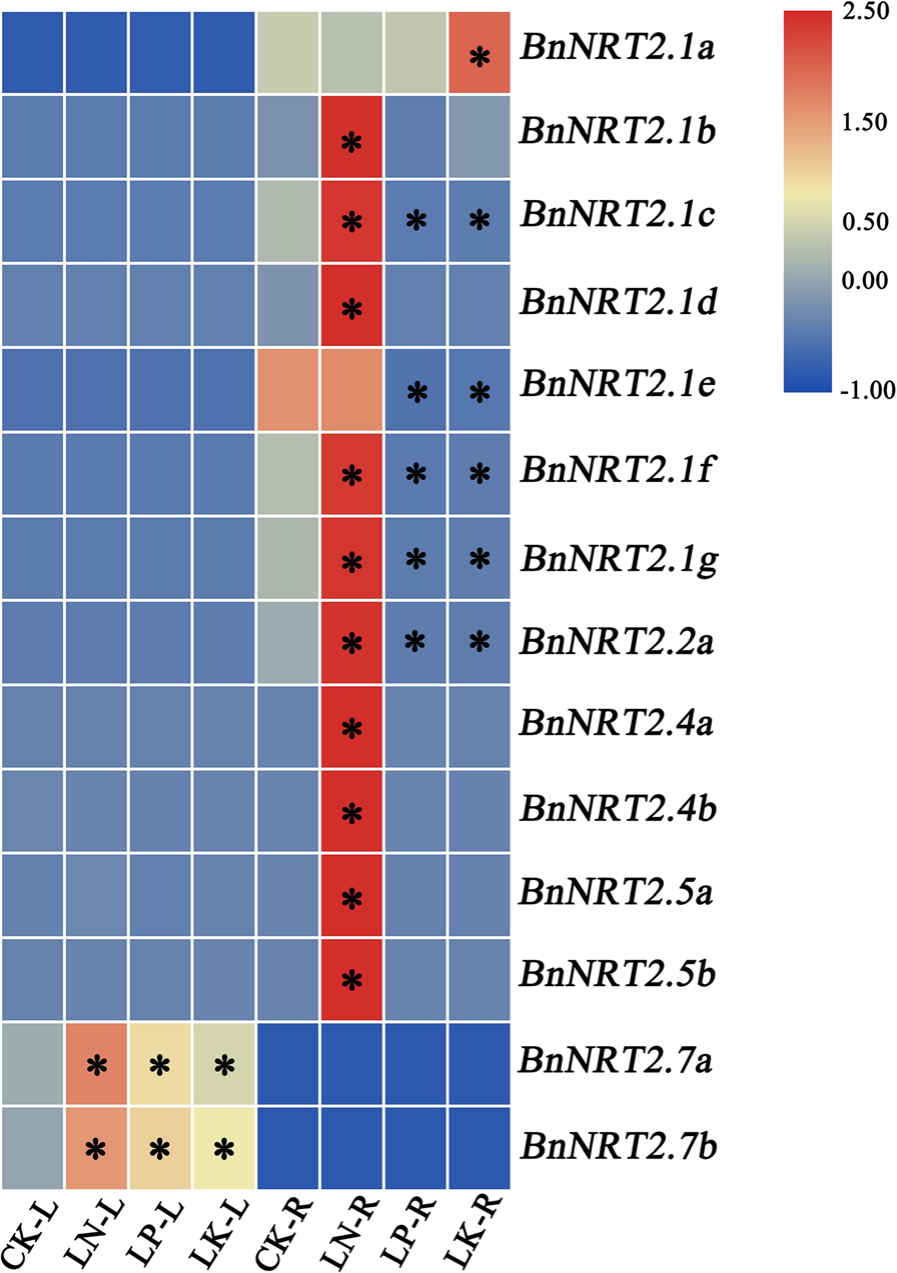
Heat maps of *BnNRT2* family genes expression profiles responding to macronutrient deficiency simulated in hydroponics. For this assay, rapeseed seedlings were cultivated in hydroponics cultures containing low N, low P or low K nutrient solution. Seedlings reared under full nutrient conditions were regarded as control samples, and *P* = 0.05 was used as the threshold to identify significant differences in expression between different conditions. Significant treatments are marked with asterisks. CK: full nutrient condition, LN: N deficiency, LP: P deficiency, LK: K deficiency, L: leaf, R: root

In this study, the expression of *BnNRT2* genes was assayed for responses to nutrient imbalances in hydroponics. To this end, rapeseed seedlings were subjected to N, phosphorus (P) and potassium (K) deficiency treatments in hydroponics cultures in order to test for responses in the expression *BnNRT2* genes to deficiencies in any of these three macronutrients. As shown in Fig. 6, most *BnNRT2.1 s* (with exception of *BnNRT2.1a* and *BnNRT2.1e*) and *BnNRT2.2a* were significantly up-regulated in roots subjected to N deficiency, but dramatically down-regulated by P or K deficiency. Interestingly, *BnNRT2.1a* appeared to be specifically up-regulated in roots in response to K deficiency, while *BnNRT2.1e* was significantly down-regulated in roots in response to P and K deficiency, but not N deficiency (Fig. 6). Moreover, *BnNRT2.4* and *BnNRT2.5* members were specifically and remarkably up-regulated by N deficiency in roots (Fig. 6). In contrast to root specific responses, leaf expressions of *BnNRT2.7 s* was significantly up-regulated by each of the tested macronutrient deficiencies (Fig. 6), suggesting that these genes might be involved in crosstalk of N, P, and K homeostasis signaling pathways.

### Responses of *BnNRT2* gene expression to drought or waterlogging stress

**Fig. 7:**
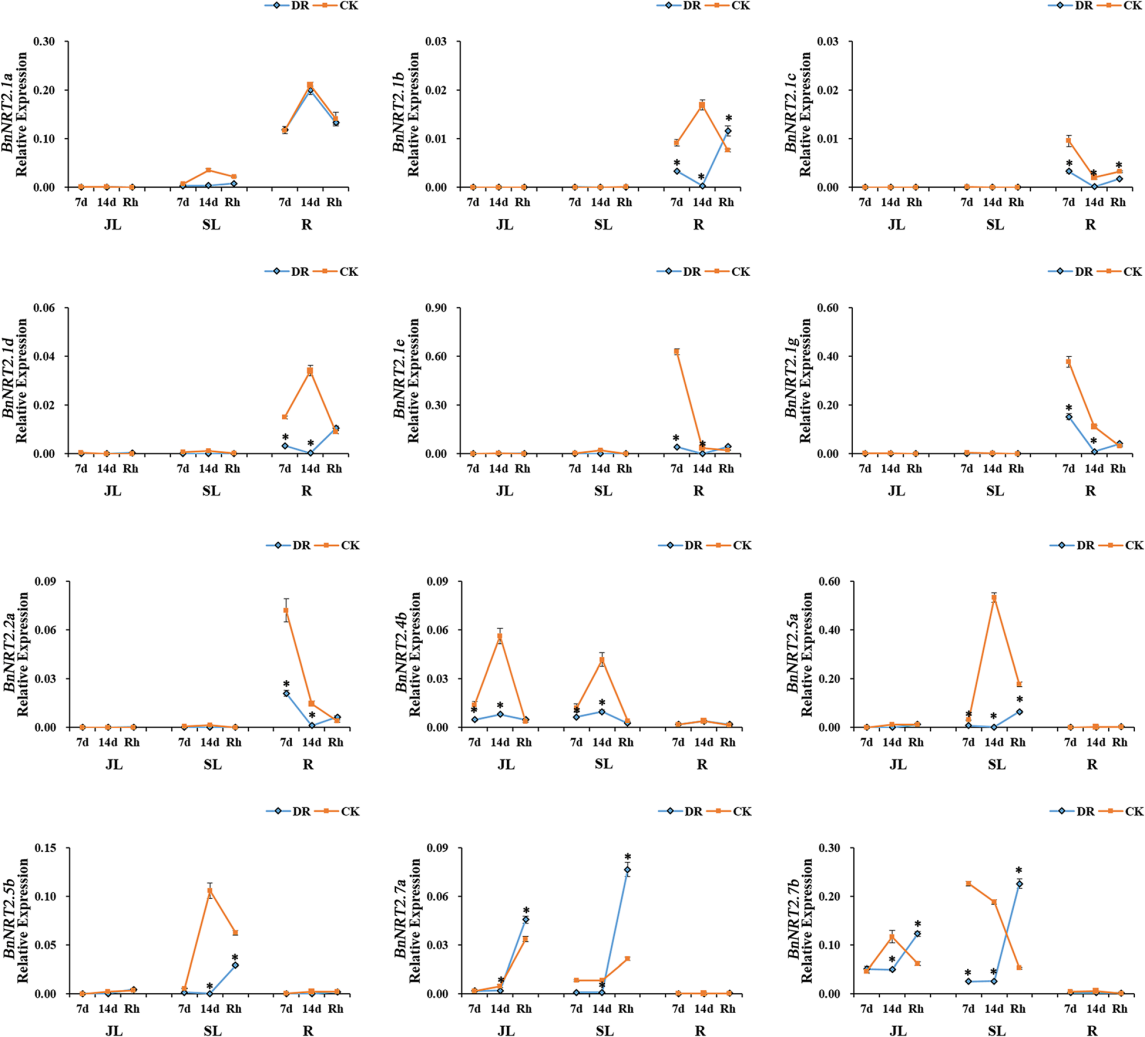
qRT-PCR assay expression profiles of *BnNRT2* family genes responding to drought stress. For this assay, rapeseed seedlings at the five leaf growth stage were subjected to drought stress for 14 day before rehydration. The tissues for qRT-PCR assays were harvested on the 7th and 14th days of drought stress, as well as, on the 7th day after rehydration. The blue line connects observations in the drought stress treatment (DR) and the orange line connects observations in the control treatment (CK). Rh: rehydration. Asterisks mark significant differences in expression between control and drought stress treatments (*P* < 0.05). JL: junior leaf, SL: senior leaf, R: root

**Fig. 8:**
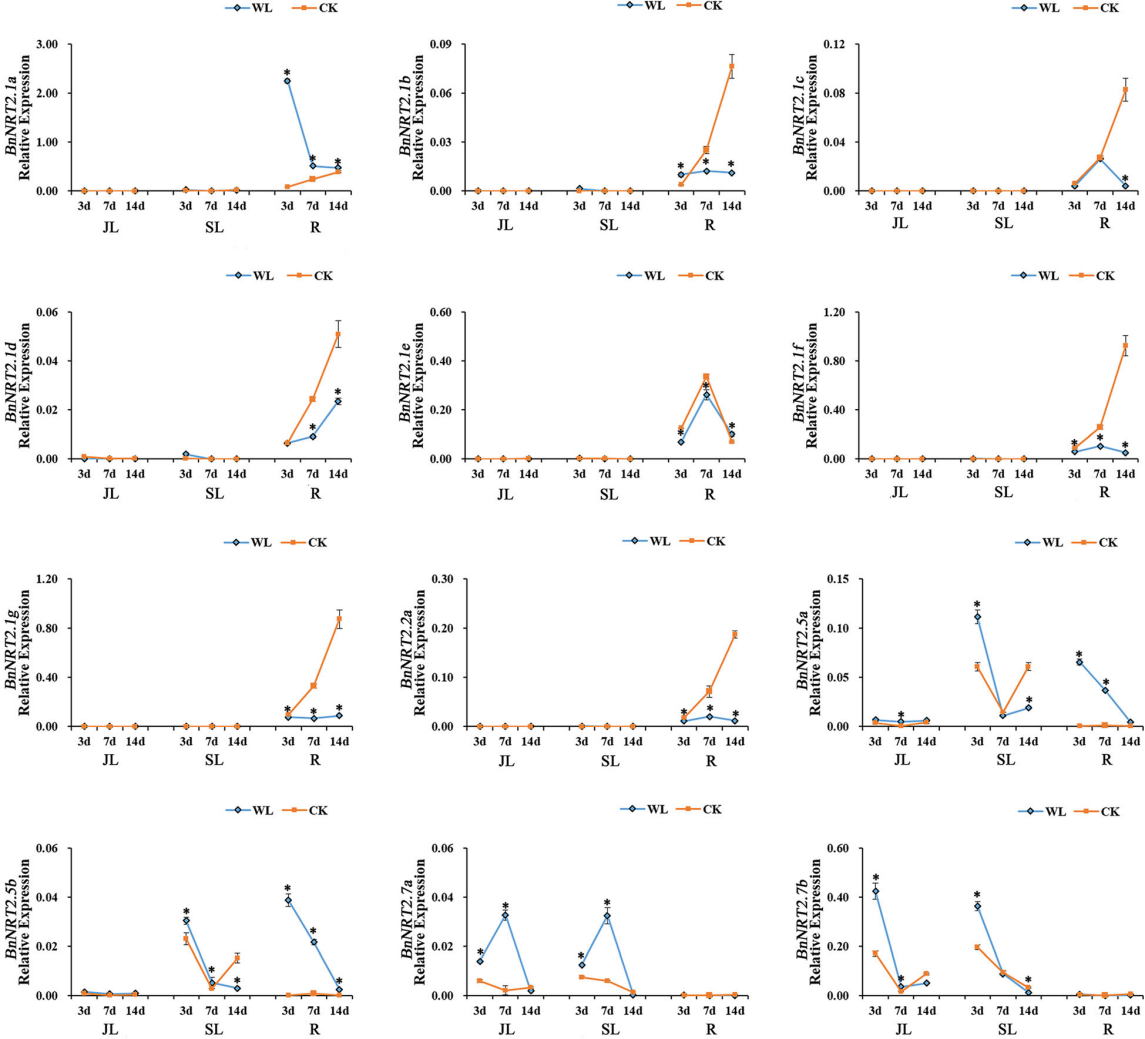
qRT-PCR assay expression profiles of *BnNRT2* family genes responding to waterlogging stress. For this assay, rapeseed seedlings at the five leaf growth stage were subjected to waterlogging stress for 14 days. Tissues for qRT-PCR assays were harvested on the 3rd, 7th and 14th days of waterlogging stress. The blue line connects observations in the waterlogging stress treatment (WL), and the orange line connects observations in the control treatment (CK). Asterisks mark significant differences in expression between control and waterlogging stress treatments (P < 0.05). JL: junior leaf, SL: senior leaf, R: root

Drought and waterlogging have extremely serious impacts on agricultural production, with severe impacts on rapeseed plants often occurring vegetative growth. In this study, drought and waterlogging stresses were simulated in pot cultures in order to investigate the expression patterns of *BnNRT2s* genes in response to these stresses. Under drought stress conditions, the transcript abundance of all *BnNRT2* genes, except *BnNRT2.1a*, dramatically decreased relative to expression in control pots over the course of 14 days of drought stress, and then restored to control levels upon rehydration (Fig. 7). Expression of *BnNRT2.1a* in drought treated pots was maintained at the levels observed in control pots (Fig. 7). At the other extreme, under waterlogging stress conditions, *BnNRT2.1a* and *BnNRT2.5 member*s were significantly up-regulated in the root, while *BnNRT2.7a* expression was significantly up-regulated in leaves (Fig. 8). At the same time, root expression of *BnNRT2.1e* in remained high in waterlogged pots and did not vary relative to expression in control pots, while, the transcript abundances of *BnNRT2.1f*, *BnNRT2.1 g* and *BnNRT2.2a* were remarkably down-regulated in roots responding to waterlogging stress (Fig. 8).

## Discussion

NRT2 proteins are common in bacteria, animals and plants. Though *NRT2* gene family members from a number of species have been described in detail, information on nitrate transporters in rapeseed, especially respond to various stresses remains limited. In this study, 17 putative *NRT2* genes were identified from the genome of rapeseed, which means that individual *Arabidopsis NRT2* genes (7 genes) may have multiple homologs in rapeseed. Indeed, that was observed in this study, but with the less expected outcome that a total of seven *BnNRT2.1* genes and two *BnNRT2.2* genes were identified based on their respective closest matches in *Arabidopsis* being the highly similar pair of *NRT2* genes, *AtNRT2.1* and *AtNRT2.2* (Fig. 1 and Table 1). The extent of *BnNRT2.1* genes in rapeseed suggests that this complex gene family might be the product of allopolyploidy or duplication events in the rapeseed evolutionary history [18], and our results indeed found that most gene duplications of the *NRT2* gene family resulted from whole-genome duplication (Table S1). In addition, results from the macronutrient deficiency experiment reported herein clearly indicate that duplication of *BnNRT2.1* genes has led to divergence of the specific roles played by members of this family in responses to varied macronutrient stress conditions. Conserved domain analysis indicated that all 17 of the putatively identified NRT2 proteins harbor the signature sequence (G-W-G/A-N/D-M/L/V-G) of NNP family proteins (Fig. 2), which are also found in other NRT2 transporters, such as the NarK protein in *E. coli* and NRT2 in *Heb. cylindrosporum* [9, 27].

Cis-acting regulatory element analysis of *BnNRT2* promoter regions revealed a great abundance of light responsive element (Fig. 3), which implies that *BnNRT2* gene expression is closely associated with photosynthesis and might be diurnally regulated. Previous studies with *Arabidopsis* have elucidated up-regulation of *AtNRT2.1* in daylight and down-regulation during the night, which can be inhibited by sucrose [28, 29]. Taking our results together with past reports supports the conclusion that, as light and sucrose regulated mediators of NO_3_^−^ uptake and transport, rapeseed *NRT*s are key members of systems coordinating carbon and N metabolism. Furthermore, the duplication of *BnNRT2.1* genes and variation in their responses to N, P and K deficiency treatments, suggests that *NRT* genes are involved in modulating multiple nutrient response pathways.

Several previous studies have suggested that limited external NO_3_^−^ supplies can promote lateral root elongation through regulation of meristematic activity in mature root tips, where, in rapeseed, cells in the meristematic region are packed more densely than in the elongation zone [30]. In *Arabidopsis*, *AtNRT2.1* and *AtNRT2.2*, two nitrate transporter genes mainly expressed in the root, play key roles in the promotion of lateral root elongation [31, 32]. Recent research has also demonstrated that knock-down of *CsNRT2.1* in cucumber strongly reduces root length and lateral root numbers under low NO_3_^−^conditions [33]. In the present study, *BnNRT2.1 s* were predominantly expressed in roots, and most were up-regulated during N deficiency stress (Figs. 5 and 6). Plus, lateral root lengths and root tips also increased in response to N deficiency (Fig. S3C, D). Therefore, we conclude that *BnNRT2.1* family members are likely involves in the regulation of the lateral root elongation under N limited conditions.

More recently, *EXO70A3* was identified as a downstream target of *PIN4*, an auxin efflux carrier, in adaptive drought responses that lead to the production of a deeper root system architecture (RSA) [34]. Analogously, a deeper RSA induced by N deficiency might be modulated by *EXO70A3*, with *NRT2.1* genes possibly involve downstream of auxin pathway related RSA modulation, which is supported by the fact that in CREs analysis, IAA responsive elements were found in the promoter sequences of *BnNRT2.1 s* (Fig. 3).

In Arabidopsis, Orsel [35] have outlined expression profiles of *NRT2* family members, with *AtNRT2.1*, *AtNRT2.4*, *AtNRT2.5* and *AtNRT2.6* being preferentially expressed in roots, *AtNRT2.7* specifically expressed in aerial parts, *AtNRT2.3* expression intermediate between the previous two profiles, and *AtNRT2.2* undetectable in either roots or shoots. Similarly, in the rapeseed expression profiles observed herein, *BnNRT2.1 s* and *BnNRT2.4 s* were mainly expressed in the root, while *BnNRT2.7 s* were detected only in aerial parts, including leaves, seeds, flowers and stems (Fig. 4b, c). On the other hand, in contrast to the observations of Orsel [35], *BnNRT2.2a* transcripts were mainly detected in roots, and no *BnNRT2.3* transcripts were detected in either roots or shoots. We did observe that *BnNRT2.5 s* were like *BnNRT2.7 s* and expressed mainly in the shoots of adult plants (Fig. 4b, c). However, in the seedlings reared in hydroponics cultures, *BnNRT2.5 s* were mainly expressed in the root (Fig. 4d). This could be an artefact of the hydroponics system, or it might suggest that *BnNRT2.5 s* expression in roots changes as the plant matures. Finally, *BnNRT2.7 s* were the only *BnNRT2*s detected in seeds (Fig. 4b), which is consistent with Chopin’s [14] finding that *AtNRT2.7* plays a specific role in nitrate accumulation in the seed.

To date, a number of *NRT2.1*-homologous genes have been cloned and characterized in higher plant species including *Arabidopsis thaliana* [10, 36], *Glycine max* [37], *Triticum aestivum* [38], *Oryza sativa* [39], *Zea mays* [40, 41], Cucumber [33], and Chinese cabbage [42], many of which have been demonstrated to be major HATS-type genes involved in root NO_3_^−^ uptake under N limitation. In accordance with previous reports, the mRNA levels of *BnNRT2.1 s* in the roots observed in this study were up-regulated with declines in N supply (Fig. 5). What’s more, recent research has identified the gene *BnaA06g04560D* (*BnNRT2.1d*) as a key factor in NUE through genomic and transcriptomic analysis experiments involving two *Brassica napus* genotypes contrasting in NUE [43]. Additionally, *BnNRT2.5* expression profiles vary between seedlings and adult plants, though expression increased with decreases in external N supply (Figs. 5 and 6). Coincidentally, previous research with *NRT2.1*, *NRT2.2*, *NRT2.4* and *NRT2.5* mutants has indicated that *AtNRT2.5*, which is expressed in the epidermis and the cortex of lateral roots in young seedlings and the minor veins of mature leaves in adult plants, works in combination with these other *NRT2*s to modulate efficient uptake of nitrate, as well as, to participate in nitrate loading into the phloem during nitrate remobilization under N-starvation conditions [13]. *BnNRT2.5 s* might perform similar functions in rapeseed as *AtNRT2.5* does in *Arabidopsis,* which could be a worthwhile subject of future investigations.

The absorption of N directly impacts crop yield, and, therefore, maintaining appropriate N fertilizer supplies is an important component of managing crop yields. However, numerous stresses, such as waterlogging, drought and nutrients deficiencies, may reduce crop yields. As expected, in the present study, it was found that waterlogging, drought, P deficiency and K deficiency suppress the expression of rapeseed *NRT2* gene members (Figs. 6, 7 and 8). Specifically, *BnNRT2.1a* was up-regulated under waterlogging stress and K deficiency conditions, *BnNRT2.7* members were up-regulated under waterlogging stress and P or K deficiencies conditions (Figs. 6 and 8), and *BnNRT2.5* members were up-regulated in roots under waterlogging stress (Fig. 8). This is consistent with the previous findings of some Pi, Fe transporters [44, 45], which were also regulated by different nutrient stresses, and it is well-documented that the cross-talk exist among ion signals when plant respond to different nutrient stresses [45–47]. The enhanced expressions of *BnNRT2s* genes under P or K deficiencies probably because the N uptake or metabolism would be influenced by these deficiencies, we speculated that these *NRT2* genes could sense the status of external P or K level. However, the synergistic regulation mechanisms of different nutrients homeostasis need to be further investigated. Additionally, the enhanced expression of *BnNRT2s* might contribute to efficient NO_3_^−^ uptake and transport of rapeseed under waterlogging stresses. Thus, it is reasonable to infer that *BnNRT2.1a*, *BnNRT2.5 s* and *BnNRT2.7 s* might play key roles in modulating N uptake and transport in rapeseed plants experiencing multiple stresses.

## Conclusions

In conclusion, 17 *NRT2* genes distributed on nine chromosomes were identified in the rapeseed genome. *BnNRT2* genes appear to play important roles during rapeseed growth and development, as indicated by their spatial and temporal expression patterns, especially under low N conditions. Notably, the expression of several *BnNRT2* increased in response to various stresses, indicating that these *NRT2* genes contribute to stress adaption through regulation of NO_3_^−^ uptake and transport. Taken together, this study included a genome-wide analysis of the rapeseed *NRT2* gene family and provides valuable information for understanding the possible functions of these genes, which could prove valuable in breeding efforts aiming to produce rapeseed plants capable yielding well in stressful environments.

## Methods

### Identification of *NRT2* genes in rapeseed

Sixteen previously reported NRT2 protein sequences, including 7 from *A.thaliana*, 3 from *Oryza sativa* (L.), 3 from *Glycine max* (L.), and 3 from *Zea mays* (L.) [39, 48, 49], were used to perform BLAST searches of the gene database for *A.thalianas*, *B.napus*, *G.max*, *O.sativa*, *Z.mays*, *T.aestivum*, *B.rape*, *B.oleracea* and *B.juncea*. Then, the 16 starting protein sequences were aligned to construct an HMM profile in HMMER (v3.1), which was used in a domain searches of the BLAST hits using an E-value threshold of 0.001. Rapeseed proteins that were returned in the BLAST search, and which contained NRT2 domains defined in the HMMER search, were finally labelled as rapeseed NRT2 proteins. The gene databases queried in this study include The Arabidopsis Information Resource (TAIR) (https://www.arabidopsis.org/) for *A. thaliana* genes and gene products; Genoscope (http://www.genoscope.cns.fr/brassicanapus/) for *B.napus* [18]; the Brassica Database (BRAD) (http://brassicadb.org/brad/) for *B.rape*, *B.oleracea* and *B.juncea* [50–52]; URGI (https://wheat-urgi.versailles.inra.fr/) for *T.aestivum* [53]; the Maize Genetics and Genomics Database (meizeGDB) (https://maizegdb.org/) for *Z.mays*; and Phytozome v12.1 (https://phytozome.jgi.doe.gov/pz/portal.html) for the other species [54]. The *NRT2* genes of rapeseed in this study were named as follows: Species abbreviation (*Bn*) + name of gene homologs in *A. thaliana* (such as *NRT2.1*) + a lowercase letter to distinguish among homologs in rapeseed for individual *A.thaliana NRT2* genes. For example, *BnNRT2.5a* and *BnNRT2.5b* are two different rapeseed homologs of *AtNRT2.5* from *Arabidopsis*.

### Bioinformatics analyses of *NRT2* genes in rapeseed

The genomic locations of rapeseed *NRT2* genes were determined in BLASTn searches using the nucleotide sequences in Genoscope (http://www.genoscope.cns.fr/blat-server/cgi-bin/colza/webBlat). The resulting coordinates were plotted in a genomic location map using MapInspect v. 2010 (http://www.softsea.com/review/MapInspect.html). The gene duplication modes were identified among rapeseed *NRT2* genes using DupGen_finder (https://github.com/qiao-xin/DupGen_finder) in *B. napus* [55]. The number of amino acids and theoretical pI were analyzed in the online Expasy software ProParam (https://web.expasy.org/cgi-bin/protparam/protparam) [56]. TargetP v 1.1 (http://www.cbs.dtu.dk/services/TargetP/) [57], WOLF PSORT (http://www.genscript.com/wolf-psort.html) [58] and ProtComp v. 9.0 (http://linux1.softberry.com/) were used to predict the subcellular localization of NRT2 proteins in rapeseed. Prediction of transmembrane helices in NRT2 proteins was performed via TMHMM 2.0 web application (http://www.cbs.dtu.dk/services/TMHMM/).

All the NRT2 protein sequences of *A.thalianas*, *B.napus*, *G.max*, *O.sativa*, *Z.mays*, *T.aestivum*, *B.rape*, *B.oleracea* and *B.juncea* were compiled in a FASTA file that was then subjected to multiple sequence alignment in ClustalW, which was then used to construct phylogenetic trees using the Neighbor-Joining method in MEGA 6.06, with the bootstrap value set at 1000, and the other parameters set to default values [59].

Exon-Intron structure information was obtained by importing each cDNA sequence (CDS) and genomic sequence of *NRT2* gene family members into the Gene Structure Display Server (GSDS 2.0) (http://gsds.cbi.pku.edu.cn/) [60]. Conserved motifs/domains were then deciphered by subjecting translated rapeseed NRT2 protein sequences to the Multiple Expectation maximization for Motif Elicitation (MEME v5.1.0) algorithm of the MEME suite of analysis tools (http://meme-suite.org/tools/meme) [61].

Promoter sequences of rapeseed *NRT2* genes were analyzed by downloading the 2000 bp region upstream from the start codon (ATG) for each gene from the Brassica Database (BRAD) (http://brassicadb.org/brad/), and then submitting these promoter regions for inspection at plantCARE (http://bioinformatics.psb.ugent.be/webtools/plantcare/html/) [62]. Putative cis-acting regulatory elements were then prepared for graphical views using TBtools v0.6.

### Plant materials and treatments

A Chinese grown rapeseed cultivar zhongshuang 11 (*B. napus* cv. ZS11), which bred by Oil Crops Research Institute of Chinese Academy of Agricultural Sciences, was the source of plant material applied in this study. Tissue specific expression analysis of *BnNRT2s* was performed in eight tissues by RNA-seq analysis, and then confirmed by rearing rapeseed plants in the field with N supplied as 180 kg/hm^2^ urea and harvesting seven tissues at several developmental stages for qRT-PCR assays, in detail, junior leaf, senior leaf and root were collected at vegetative growth stage (namely over-wintering stage), flower and stem were collected at flowering stage, then seed and pericarp were collected at reproductive growth stage. Three independent biological replicates were included for tissue specific analysis.

To investigate possible responses of *BnNRT2s* to nutrient deficiency, 7-day-old seedlings were exposed to low nitrogen (LN), low phosphorus (LP), and low potassium (LK) conditions for 14 days, in which time nutrient deficiency symptoms became evident. In more detail, nutrient deficiency conditions were simulated in hydroponics cultures with the base solution being 1/2 strength modified Hoagland nutrient solution, containing 2.5 mM Ca (NO_3_)_2_, 2.5 mM KNO_3_, 1 mM NH_4_NO_3_, 0.25 mM K_2_SO_4_, 0.5 mM KH_2_PO_4_, 1 mM MgSO_4_, 0.08 mM EDTA-Fe, 0.02 mM H_3_BO_4_, 4.5 μM MnCl_2_, 0.3 μM ZnSO_4_, 0.16 μM CuSO_4_, and 0.16 μM (NH_4_)_6_Mo_7_O_24_. Control solution contained 9.5 mM N, 0.5 mM P and 3.5 mM K. The nutrient deficiency treatment solutions were the same as the control solution, except LN contained 0.475 mM N (5% of control N), LP contained 10 μM P (2% control P), and LK contained 10 μM K (0.29% of control K). Plants were grown in a 24 °C/20 °C day/night temperature regime with a 14-h/10-h light/dark photoperiod. Each treatment had four independent biological replicates. Nutrient (N, P, K)-deficient rapeseed leaves and roots were separately sampled for further RNA extraction and qRT-PCR analysis.

Additionally, *BnNRT2* responses to N availability were performed in a field experiment conducted in Huanggang city of Hubei province. Experimental plots were planted with the rapeseed genotype ZS11 and subjected to routine management practices. Nitrogen was supplied to plots as urea (46% N) at the rates of 0, 45, or 180 kg/hm^2^. Each treatment had three independent biological replicates. Seven tissues at several developmental stages under different N rates were sampled for qRT-PCR assays, in detail, junior leaf, senior leaf and root were collected at vegetative growth stage (namely over-wintering stage), flower and stem were collected at flowering stage, then seed and pericarp were collected at reproductive growth stage.

Moreover, drought and waterlogging stress were also tested in pot cultures set up in accordance with previous research [63]. For drought stress observations weighed pots were maintained with water content ranging between 25 to 35% for a drought treatment, and between 65 to 75% in control pots. Rapeseed seedlings at the five leaf growth stage were subjected to drought stress for 14 day before rehydration. Rapeseed junior leaf, senior leaf and root were separately sampled after 7 days, 14 days drought treatment and rehydration for 7 days. Rapeseed seedlings at the five leaf growth stage also were subjected to waterlogging stress for 14 days. Waterlogging stress, meanwhile, was conducted by keeping the water surface 1 ~ 2 cm above the soil surface, with the control pots being the same as those used in the observations of drought stress. Tissues (junior leaf, senior leaf and root) for qRT-PCR assays were harvested on the 3rd, 7th and 14th days of waterlogging stress. Each treatment in this pot experiment had three independent biological replicates.

### RNA-seq analysis and quantitative real time RT-PCR (qRT-PCR)

For tissue specific analysis of rapeseed *NRT2* family genes by RNA-seq technique, the detail of generation of RNA-Seq data had been described in detail previously [64, 65], and the publically RNA-seq dataset of various tissues collected from different rapeseed growth stages were deposited in a public repository (SRA accession: PRJNA474576, 65] and applied in this study, the Fragments per kilobase of exon per million fragments mapped (FPKM) was used to calculate gene expression levels, and a heat map of gene expression profiles (log2) of *BnaNRT2* genes was generated using pheatmap package in R software after data normalization (https://cran.r-project.org/web/packages/pheatmap/).

For verify the tissue specific expression and further analyze the differential expression profiles of rapeseed *NRT2* family genes under various stresses, the total RNA of each harvested samples were extracted using the HiPure Plant RNA Mini Kit according to manufacturer instructions (Magen, Guangzhou, China). Then, samples were treated with RNase-free DNaseI (Invitrogen, Grand Island, NY,USA) before being included as templates for cDNA synthesis using the PrimeScript™ RT reagent Kit with gDNA Eraser (Perfect Real Time) (TaKaRa, Tokyo, Japan). Following cDNA synthesis, the relative expression of rapeseed *NRT2* gene family members was detected by qRT-PCR using the SYBR® Premix Ex Taq™ II kit (Tli RNaseH Plus) (TaKaRa, Tokyo, Japan) and a CFX96™ Real-Time PCR Detection System (BIO-RAD,USA). The PCR program was run as follows: 95 °C for 1 min, followed by 40 cycles of 95 °C for 15 s, 60 °C for 15 s, and 72 °C for 30 s. The PCR reaction volume was 20 μL in total, which included 2 μL diluted cDNA, 10 μL SYBR Premix Ex Taq II (Tli RNaseH Plus)(2×), 0.6 μL primers and 6.8 μL RNAfree water (TaKaRa, Tokyo, Japan). The *Cq* value of candidate genes were normalized relative to the *Cq* value of reference gene *act-7*, and relative expression was calculated using the 2 ^-ΔCq^ method. The specific primers for rapeseed *NRT2* genes (Table S3) and the reference gene *act-7* were designed in Primer-NCBI (https://www.ncbi.nlm.nih.gov/tools/primerblast/index.cgi?LINK_LOC=BlastHome), and confirmed by Melt curve analysis, with the reaction program as follows: 95 °C for 10 s, 65 °C to 95 °C for 5 s (+ 0.5 °C per cycle).

### Statistical analyses

All means and standard errors of data in the present study were calculated in Microsoft Excel 2010. All the comparisons between control and stress treatment in this study were performed using analysis of variance (ANOVA) method in SPSS statistics 25 with *P* values < 0.05 considered statistically significant.

## Supplementary information

**Additional file 1: Figure S1.** Physical map of *NRT2* family genes in the genome of rapeseed (*Brassica napus* L.)

**Additional file 2: Figure S2.** Membrane-spanning regions predicted for NRT2 family proteins in rapeseed (*Brassica napus* L.).

**Additional file 3: Figure S3.** Phenotypic and physiological changes of rapeseed plants grown under different N supply treatments. (A) Phenotypes and biomasses of rapeseed plants grown under different N supply treatments in the field plots supplied with 180, 45 or 0 kg/hm^2^ N. (B) Biomasses of rapeseed plants grown under different N supply treatments in the field plots supplied with 180, 45 or 0 kg/hm^2^ N. (C) Phenotypes of rapeseed roots grown under different N supply treatments (CK: 9.5 mM N, LN: 0.475 mM N) in hydroponics. (D) Lateral root length and root tips of rapeseed plants grown under different N supply treatments (CK: 9.5 mM N, LN: 0.475 mM N) in hydroponics.

**Additional file 4: Table S1.** The duplicated rapeseed *NRT2* gene pairs identified in this study.

**Additional file 5: Table S2.** The comparison of identity between the BnNRT2 protein members.

**Additional file 6: Table S3.** Specific primers of rapeseed *NRT2* genes used in qRT-PCR assays.

## Data Availability

The Arabidopsis, Wheat and Maize NRT2 protein sequences were collected from TAIR (http://www.arabidopsis.org), URGI (https://wheat-urgi.versailles.inra.fr/) and meizeGDB (https://maizegdb.org/) database. The Rice and Soybean NRT2 protein sequences were collected from Phytozome v12.1 (https://phytozome.jgi.doe.gov/pz/portal.html). And the NRT2 protein sequences of *B.rape*, *B.oleracea* and *B.juncea* were collected from BRAD (http://brassicadb.org/brad/). The genome and protein sequences of *B.napus* were downloaded from Genoscope (http://www.genoscope.cns.fr/brassicanapus/). The RNA sequencing data used in this study were deposited in the NCBI SRA database (SRA accession: PRJNA474576). All data generated or analyzed during this study were included in this published article and its supplementary information files. The materials are available upon request by contacting the corresponding author.

## Abbreviations

N: nitrogen
NUE: nitrogen use efficiency
NO_3_^−^: nitrate
LATS: low-affinity transport system
HATS: high-affinity transport system
NRT1: nitrate transporter 1
NRT2: nitrate transporter 2
NNP: nitrate/nitrite porter family
MFS: major facilitator superfamily
CREs: cis-acting regulatory elements
TFs: transcription factors
P: phosphorus
K: potassium
FPKM: Fragments per kilobase of exon per million fragments mapped
