# Supplementary figures and images for: Genome-wide identification and analysis of high-affinity nitrate transporter 2 (NRT2) family genes in rapeseed (Brassica napus L.) and their responses to various stresses
Source: BMC Plant Biol. 2020 Oct 9;20:464. doi: 10.1186/s12870-020-02648-1 (PMC7547492; doi:10.1186/s12870-020-02648-1)

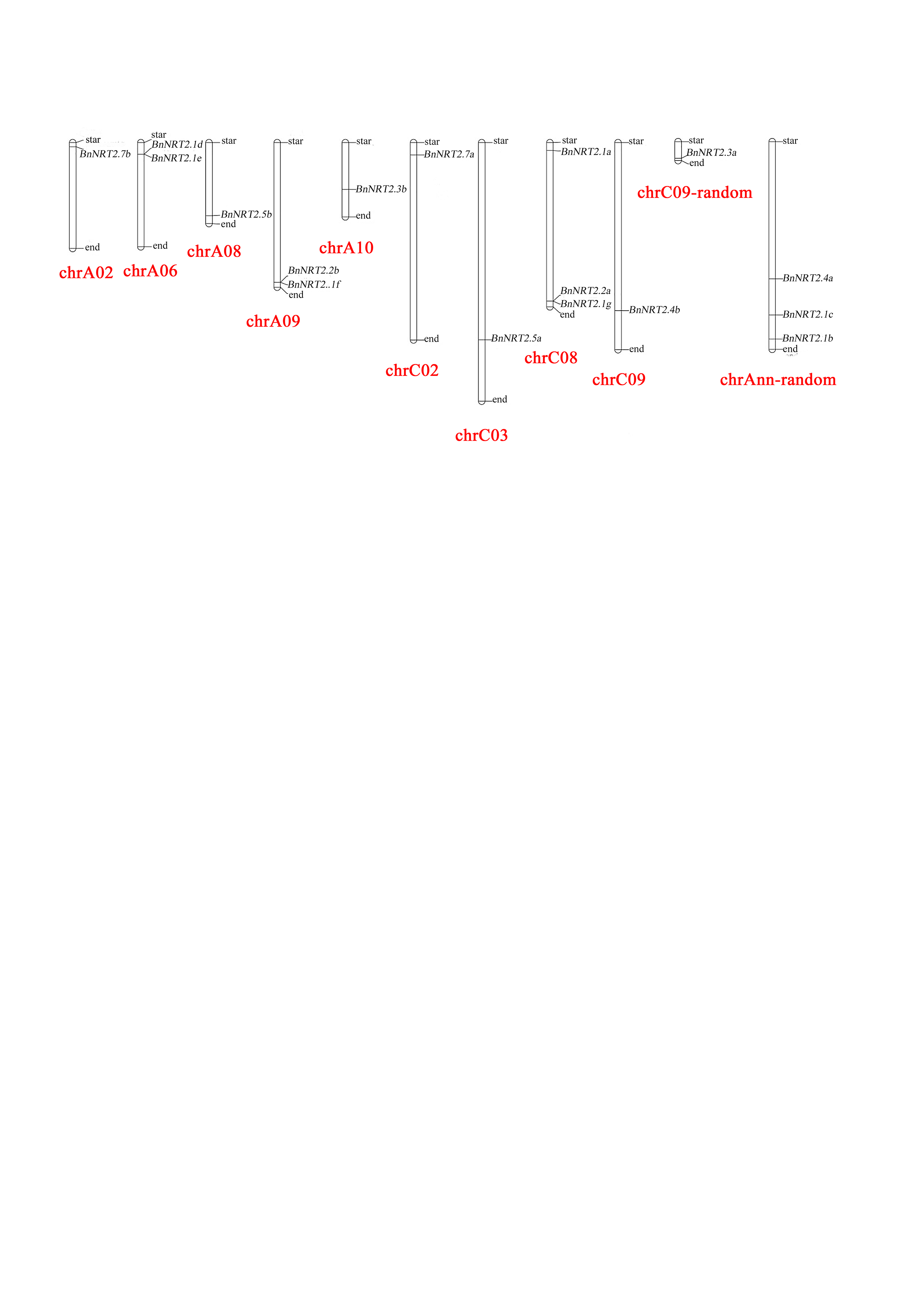

Supplement: Supplementary file 1 — Additional file 1: Figure S1. Physical map of NRT2 family genes in the genome of rapeseed (Brassica napus L.) [file 12870_2020_2648_MOESM1_ESM.tif]

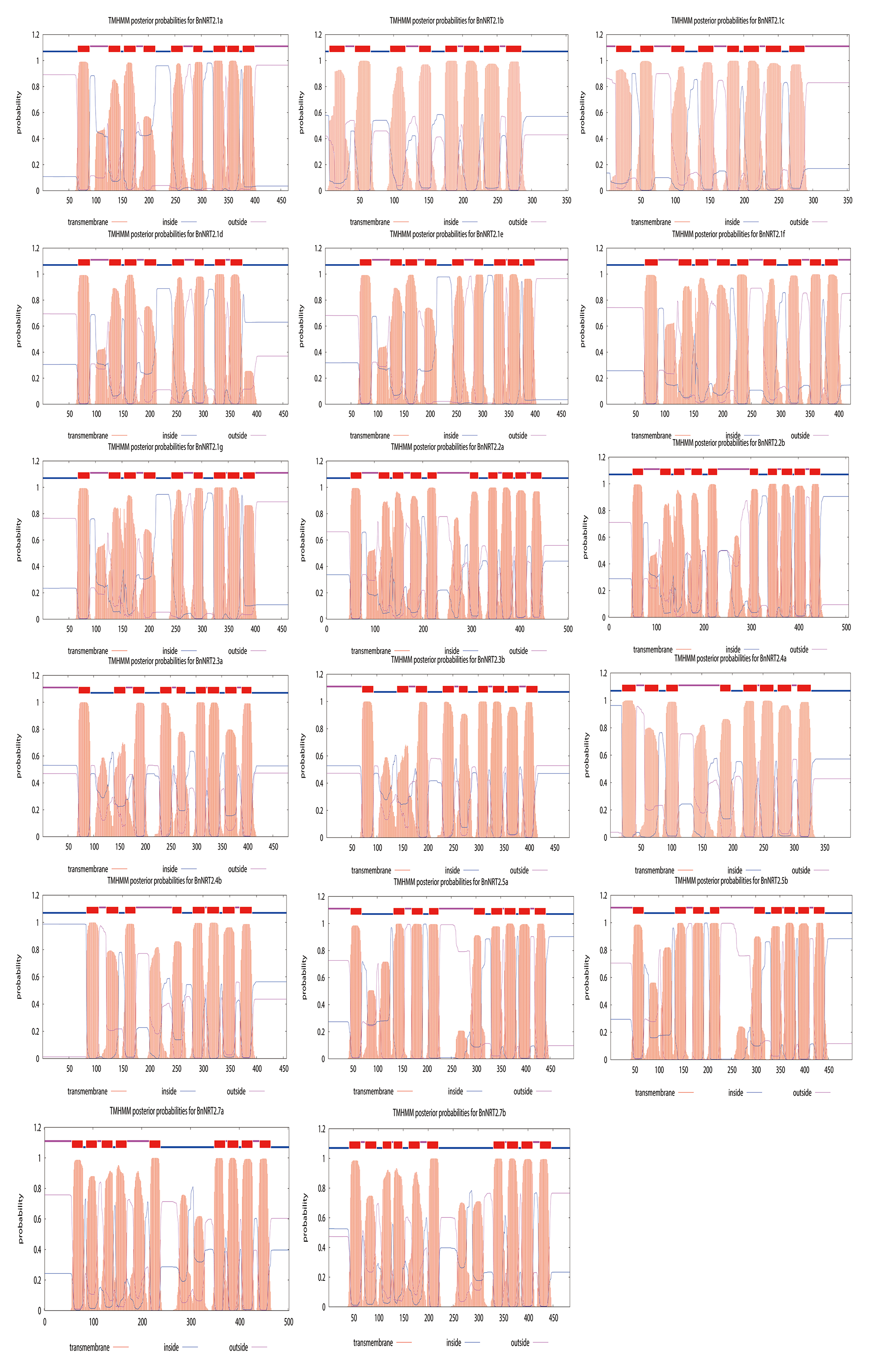

Supplement: Supplementary file 2 — Additional file 2: Figure S2. Membrane-spanning regions predicted for NRT2 family proteins in rapeseed (Brassica napus L.). [file 12870_2020_2648_MOESM2_ESM.tif]

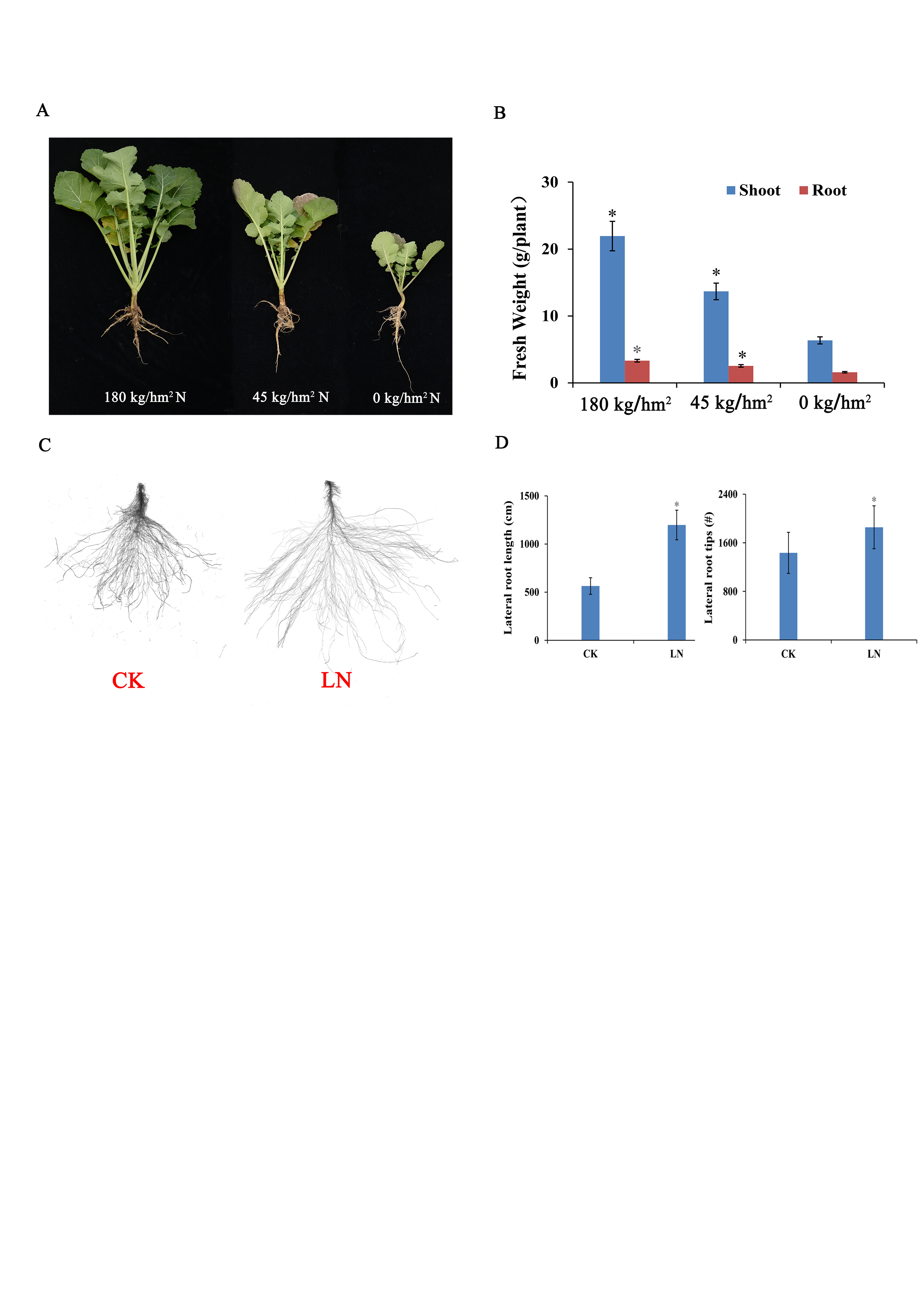

Supplement: Supplementary file 3 — Additional file 3: Figure S3. Phenotypic and physiological changes of rapeseed plants grown under different N supply treatments. (A) Phenotypes and biomasses of rapeseed plants grown under different N supply treatments in the field plots supplied with 180, 45 or 0 kg/hm2 N. (B) Biomasses of rapeseed plants grown under different N supply treatments in the field plots supplied with 180, 45 or 0 kg/hm2 N. (C) Phenotypes of rapeseed roots grown under different N supply treatments (CK: 9.5 mM N, LN: 0.475 mM N) in hydroponics. (D) Lateral root length and root tips of rapeseed plants grown under different N supply treatments (CK: 9.5 mM N, LN: 0.475 mM N) in hydroponics. [file 12870_2020_2648_MOESM3_ESM.tif]
