# Supplementary material for: Genome-wide identification and analysis of high-affinity nitrate transporter 2 (NRT2) family genes in rapeseed (Brassica napus L.) and their responses to various stresses
Source: BMC Plant Biol. 2020 Oct 9;20:464. doi: 10.1186/s12870-020-02648-1 (PMC7547492; doi:10.1186/s12870-020-02648-1)
Supplement: Supplementary file 4 — Additional file 4: Table S1. The duplicated rapeseed NRT2 gene pairs identified in this study. [file 12870_2020_2648_MOESM4_ESM.docx]

| No. | Duplicated gene pairs | | Duplication type |
| --- | --- | --- | --- |
| 1 | *BnNRT2.7b* | *BnNRT2.7a* | wgd |
| 2 | *BnNRT2.1d* | *BnNRT2.2b* | wgd |
| 3 | *BnNRT2.1d* | *BnNRT2.3b* | wgd |
| 4 | *BnNRT2.1d* | *BnNRT2.1a* | wgd |
| 5 | *BnNRT2.1d* | *BnNRT2.2a* | wgd |
| 6 | *BnNRT2.5b* | *BnNRT2.5a* | wgd |
| 7 | *BnNRT2.2b* | *BnNRT2.2a* | wgd |
| 8 | *BnNRT2.2b* | *BnNRT2.1a* | wgd |
| 9 | *BnNRT2.3b* | *BnNRT2.4b* | wgd |
| 10 | *BnNRT2.1a* | *BnNRT2.2a* | wgd |
| 11 | *BnNRT2.4a* | *BnNRT2.4b* | transposed |
| 12 | *BnNRT2.1c* | *BnNRT2.2b* | transposed |
| 13 | *BnNRT2.1b* | *BnNRT2.2b* | transposed |
| 14 | *BnNRT2.3a* | *BnNRT2.3b* | transposed |

**Table S1** The duplicated rapeseed *NRT2* gene pairs identified in this study

Note: wgd represents whole-genome duplication
