# Supplementary material for: Genome-wide identification and analysis of high-affinity nitrate transporter 2 (NRT2) family genes in rapeseed (Brassica napus L.) and their responses to various stresses
Source: BMC Plant Biol. 2020 Oct 9;20:464. doi: 10.1186/s12870-020-02648-1 (PMC7547492; doi:10.1186/s12870-020-02648-1)
Supplement: Supplementary file 5 — Additional file 5: Table S2. The comparison of identity between the BnNRT2 protein members. [file 12870_2020_2648_MOESM5_ESM.docx]

|  | **BnNRT**  **2.1a** | **BnNRT**  **2.1b** | **BnNRT**  **2.1c** | **BnNRT**  **2.1d** | **BnNRT**  **2.1e** | **BnNRT**  **2.1f** | **BnNRT**  **2.1g** | **BnNRT 2.2a** | **BnNRT**  **2.2b** | **BnNRT**  **2.3a** | **BnNRT**  **2.3b** | **BnNRT**  **2.4a** | **BnNRT**  **2.4b** | **BnNRT**  **2.5a** | **BnNRT**  **2.5b** | **BnNRT**  **2.7a** | **BnNRT**  **2.7b** |
| --- | --- | --- | --- | --- | --- | --- | --- | --- | --- | --- | --- | --- | --- | --- | --- | --- | --- |
| **BnNRT2.1a** | 100.0% |  |  |  |  |  |  |  |  |  |  |  |  |  |  |  |  |
| **BnNRT2.1b** | 54.0% | 100.0% |  |  |  |  |  |  |  |  |  |  |  |  |  |  |  |
| **BnNRT2.1c** | 51.1% | 93.8% | 100.0% |  |  |  |  |  |  |  |  |  |  |  |  |  |  |
| **BnNRT2.1d** | 95.5% | 51.7% | 51.1% | 100.0% |  |  |  |  |  |  |  |  |  |  |  |  |  |
| **BnNRT2.1e** | 95.9% | 52.1% | 51.9% | 97.6% | 100.0% |  |  |  |  |  |  |  |  |  |  |  |  |
| **BnNRT2.1f** | 74.5% | 43.3% | 45.5% | 76.7% | 76.3% | 100.0% |  |  |  |  |  |  |  |  |  |  |  |
| **BnNRT2.1g** | 94.4% | 50.6% | 53.1% | 95.0% | 95.9% | 76.9% | 100.0% |  |  |  |  |  |  |  |  |  |  |
| **BnNRT2.2a** | 72.6% | 59.0% | 59.3% | 73.6% | 73.4% | 71.7% | 73.7% | 100.0% |  |  |  |  |  |  |  |  |  |
| **BnNRT2.2b** | 74.0% | 60.5% | 59.1% | 73.8% | 73.8% | 70.6% | 74.1% | 94.5% | 100.0% |  |  |  |  |  |  |  |  |
| **BnNRT2.3a** | 52.9% | 47.8% | 46.7% | 51.3% | 49.7% | 47.2% | 51.3% | 56.3% | 57.7% | 100.0% |  |  |  |  |  |  |  |
| **BnNRT2.3b** | 51.9% | 47.5% | 46.9% | 49.3% | 49.9% | 47.6% | 51.3% | 57.3% | 57.5% | 97.5% | 100.0% |  |  |  |  |  |  |
| **BnNRT2.4a** | 47.6% | 72.5% | 71.4% | 46.6% | 47.9% | 43.2% | 46.3% | 57.1% | 57.7% | 49.0% | 49.0% | 100.0% |  |  |  |  |  |
| **BnNRT2.4b** | 51.1% | 62.5% | 61.6% | 48.9% | 50.0% | 45.6% | 48.8% | 59.5% | 60.1% | 50.2% | 50.2% | 82.2% | 100.0% |  |  |  |  |
| **BnNRT2.5a** | 49.3% | 39.9% | 39.8% | 48.9% | 49.6% | 46.0% | 49.7% | 57.1% | 56.9% | 46.3% | 46.6% | 42.7% | 46.4% | 100.0% |  |  |  |
| **BnNRT2.5b** | 49.4% | 40.2% | 40.1% | 49.1% | 49.8% | 46.0% | 49.9% | 57.1% | 56.9% | 46.5% | 46.8% | 43.0% | 46.8% | 98.8% | 100.0% |  |  |
| **BnNRT2.7a** | 36.6% | 26.9% | 27.3% | 37.5% | 37.4% | 38.4% | 36.7% | 42.3% | 42.1% | 36.2% | 35.7% | 29.3% | 32.0% | 47.8% | 47.6% | 100.0% |  |
| **BnNRT2.7b** | 36.5% | 28.2% | 28.3% | 37.7% | 37.3% | 38.1% | 36.5% | 43.6% | 42.8% | 36.2% | 35.4% | 30.0% | 32.9% | 48.5% | 48.2% | 92.5% | 100.0% |

**Table S2** The comparison of identity between the BnNRT2 protein members
