# Supplementary material for: Genome-wide identification and analysis of high-affinity nitrate transporter 2 (NRT2) family genes in rapeseed (Brassica napus L.) and their responses to various stresses
Source: BMC Plant Biol. 2020 Oct 9;20:464. doi: 10.1186/s12870-020-02648-1 (PMC7547492; doi:10.1186/s12870-020-02648-1)
Supplement: Supplementary file 6 — Additional file 6: Table S3. Specific primers of rapeseed NRT2 genes used in qRT-PCR assays. [file 12870_2020_2648_MOESM6_ESM.docx]

| **Gene Name** | **Forward Primer** | **Reverse Primer** |
| --- | --- | --- |
| ***BnNRT2.1a*** | CACTTGAAGCTCCACACAGCC | AAAGTAAGTGGCTGCAATGTCG |
| ***BnNRT2.1b*** | CGCAAACACCCTCGTCACAG | TGATTCCAAGGGCTCGTCTG |
| ***BnNRT2.1c*** | ATGCTTCGGAGTGGAACGAG | CCCTCTGACTTGGCGTTCTC |
| ***BnNRT2.1d*** | CGCCGTTACAAACTACAGGACT | GCAGTGTGAAGCTTCAAGTGAAAT |
| ***BnNRT2.1e*** | GGCATCATCTCAGTTGCTTGT | CTTGGCGTTCTCAGCAAACC |
| ***BnNRT2.1f*** | CCGCTTGTGGAGCTACCTTT | AACATTTCGGATATCCCGGTT |
| ***BnNRT2.1g*** | AAGGCGCTGTGGTCAAGAT | AGTTTGTGACGGCGTACCAC |
| ***BnNRT2.2a*** | GGAGGAAACTTCGGGTCAGG | GCTCCGTCGTAACTGTGGAA |
| ***BnNRT2.2b*** | AATACTGGATGAGCACTATGTTCACC | GGCATGAGCAACTGCGTTATC |
| ***BnNRT2.3a*** | GTGCTAATTCACTTCCCATAGCC | CGAGACGAATGGTGCAACCT |
| ***BnNRT2.3b*** | TGGTCAAGTTTTCTGGTTCGC | CATTCCGAAGCTGGCTGCTA |
| ***BnNRT2.4a*** | GCAATGGGGGAGCATGTTTT | TCAGGTCCAGAAGCCACGAA |
| ***BnNRT2.4b*** | CAGAGAATTCCCGGAGACCA | TGAGATCTGGTCACCACCAC |
| ***BnNRT2.5a*** | TCTCGAACCACCACCACTCG | GAGAAGAGGCGGAACGTTGT |
| ***BnNRT2.5b*** | CGGTGTACTTCACGGCTGG | ATACCATTAGCCGAGCCCAC |
| ***BnNRT2.7a*** | CACGCCCGACAGTAACAGAA | ACGCTCTGTAGTCTCCGAGT |
| ***BnNRT2.7b*** | AGACACACCCGACGGTAAGA | TCCGTCGAGAAGGATTCGGA |

**Table S3** Specific primers of rapeseed *NRT2* genes used in qRT-PCR assays
